# Locked and Loaded: Mechanisms Regulating Natural Killer Cell Lytic Granule Biogenesis and Release

**DOI:** 10.3389/fimmu.2022.871106

**Published:** 2022-04-26

**Authors:** Hyoungjun Ham, Michael Medlyn, Daniel D. Billadeau

**Affiliations:** ^1^ Division of Oncology Research, Mayo Clinic, Rochester, MN, United States; ^2^ Department of Immunology College of Medicine, Mayo Clinic, Rochester, MN, United States

**Keywords:** natural killer cells, lytic granule, degranulation, primary immunodeficiency, cytotoxicity

## Abstract

NK cell-mediated cytotoxicity is a critical element of our immune system required for protection from microbial infections and cancer. NK cells bind to and eliminate infected or cancerous cells *via* direct secretion of cytotoxic molecules toward the bound target cells. In this review, we summarize the current understanding of the molecular regulations of NK cell cytotoxicity, focusing on lytic granule development and degranulation processes. NK cells synthesize apoptosis-inducing proteins and package them into specialized organelles known as lytic granules (LGs). Upon activation of NK cells, LGs converge with the microtubule organizing center through dynein-dependent movement along microtubules, ultimately polarizing to the cytotoxic synapse where they subsequently fuse with the NK plasma membrane. From LGs biogenesis to degranulation, NK cells utilize several strategies to protect themselves from their own cytotoxic molecules. Additionally, molecular pathways that enable NK cells to perform serial killing are beginning to be elucidated. These advances in the understanding of the molecular pathways behind NK cell cytotoxicity will be important to not only improve current NK cell-based anti-cancer therapies but also to support the discovery of additional therapeutic opportunities.

## 1 Introduction

Natural Killer (NK) cells are cytotoxic lymphocytes of the innate immune system that provide immune surveillance and first-line defense against microbial infections and tumors (1–4). Human NK cells compose 5-15% of circulating peripheral blood lymphocytes, but also present wide tissue distribution with varying numbers and sub-populations (5). Although NK cells modulate immune responses by producing a variety of inflammatory cytokines and chemokines (2, 6), NK cell cytotoxicity is the most critical function required for the ultimate clearance of tumorous, infected, or stressed cells. Like other immune cells, the overall activation and maturation of circulating NK cells is affected by inflammatory cytokines and chemokines (7, 8). However, the recognition and binding of NK cells to tumorous or unhealthy “non-self” target cells is the major driver that induces NK cell cytotoxicity (9). A wide range of activating and inhibitory receptors are expressed on the surface of NK cells, and upon binding to its target cell, a balance of signals from engaged activating and inhibitory receptors determines the NK cell response. In this way, NK cells can identify “non-self” cells to kill, while maintaining self-tolerance. Since NK cells rely on germ-line encoded NK receptors without any DNA rearrangement, they are categorized as innate members of the immune system. However, NK cells also present advanced immune functions like T and B cells, in which they present memory-like responses against specific antigens and certain activating cytokines (2).

The initial tethering of an NK cell to a target cell is mediated by adhesion molecules including selectins and integrins expressed on the NK cell surface (1, 10). Upon activation, the NK cell establishes a specialized interface with the target cell known as the cytotoxic synapse (CS), which is mediated by increased affinity interaction of integrins with their ligands expressed on the target cells. The CS is further strengthened as the actin cytoskeleton at the CS is reorganized and more integrins are recruited to the CS. Ultimately, NK cells secrete preformed secretory lysosomes called lytic granules (LGs) directly toward bound target cells, a process known as cell-mediated cytotoxicity (1–4). However, NK cells can also induce death receptor-mediated apoptosis of target cells (4, 11, 12). NK cells express death receptor ligands including FasL (CD95L) and TNF-related apoptosis-inducing ligand (TRAIL) (13). Engagement of these ligands with Fas (CD95) and TRAIL-R1/-R2, respectively, on the target cells can induce target cell apoptosis. Additionally, NK cells secrete biologically active extracellular vesicles (EVs) that contain cytotoxic proteins like perforin and granzymes and other immune modulatory molecules (14–17). These secreted vesicles seem to have immune regulatory functions and present anti-tumor effects. For a discussion of the similarities and distinctions between the LGs and NK-EVs regarding composition and molecular processes, the reader is referred to a recent excellent review (18). In line with this, both CD8^+^ cytotoxic T lymphocytes (CTLs) and NK cells were also found to secrete cytotoxic supramolecular attack particles (SMAP) composed of thrombospondin-1 (TSP-1), perforin, and granzyme B (19, 20). SMAP is distinct from extracellular vesicles because it exists in a membrane-less protein complex in which perforin and granzyme B are contained within a glycoprotein TSP-1 shell. Future studies will be required to elucidate the detailed characteristics of these extracellular vesicles and protein complexes including the physiological functions and molecular pathways behind their synthesis and secretion as well as their mechanism(s) of action. Additionally, elucidating how NK cells protect themselves from the cytotoxic effects of NK-EVs and SMAP will be an interesting and important area for future research.

Many current approaches in cancer immunotherapy rely on the cytotoxic activities of NK cells (4, 21–23). Several cytokine and checkpoint inhibitor therapies are designed to enhance the cytotoxicity of NK cells against tumors. In the field of adoptive transfer and chimeric antigen receptor (CAR) therapies, NK cells are thought to possess several advantages compared to CTLs: 1) readiness for cytotoxicity without pre-activation and clonal expansion, 2) relatively short lifespan, 3) lack of requirement for antigen specificity targeting tumor cells, and 4) lack of requirement to match major histocompatibility complex (MHC) molecules expressed on the target cells. In addition, antibody-based therapies against tumor-specific antigens can induce antibody-dependent cellular cytotoxicity (ADCC) by NK cells, since low affinity Fc receptor CD16 (FcγRIIIA) is a major activating receptor on NK cells. These examples of utilizing NK cell-mediated cytotoxicity in cancer therapies highlight the importance of better understanding the mechanisms behind the cellular cytotoxicity of NK cells.

In this review, we will summarize the current understanding of the mechanisms of NK cell-mediated cytotoxicity from LG biogenesis to the degranulation process. For updates on additional modes of NK cytotoxicity or other NK cell functions and biology, the reader is referred to other excellent reviews (1–4, 10).

## 2 Biogenesis of Lytic Granules

Cell-mediated cytotoxicity of NK cells and CTLs is achieved by the directed release of cytolytic granules toward bound target cells. Lytic granules (LGs) are a specialized subset of lysosomes which contains both lysosomal and secretory proteins that are usually compartmentalized in separate organelles in most other cell types (1, 24, 25). Therefore, LGs are also referred to as secretory lysosomes. In the case of CTLs, resting unstimulated cells do not express LGs (26, 27). Only upon T cell receptor engagement, CTLs initiate biosynthesis of electron-dense LGs. On the contrary, NK cells constitutively express LGs, thereby enabling NK cells to be primed for killing without any prior sensitization.

Secretory lysosomes are like lysosomes in that both have similar morphology and contain an acidic environment with a pH ranging from 5.1-5.4 (28). Like lysosomes, secretory lysosomes also contain proteins with hydrolytic and degradative functions like acid hydrolases and contain common lysosomal soluble (including cathepsins) and transmembrane (including lysosome-associated membrane protein [LAMP]) proteins. However, secretory lysosomes are distinguished from lysosomes by the following characteristics. First, secretory lysosomes contain additional specialized cell-type-specific components. Most cell types containing secretory lysosomes are hematopoietic lineage cells, but secretory lysosomes are also found in melanocytes and endothelial cells. In melanocytes, the secreted contents include melanin protein which is responsible for the pigmentation of skin. On the other hand, the LGs of NK cells and CTLs are mainly composed of pore-forming and apoptosis-inducing molecules such as perforin, granzymes, granulysin, and Fas ligand. Another major distinction is that although both organelles are the endpoint of endocytic pathways, secretory lysosomes undergo additional secretion processes under certain stimulatory conditions. The secretion process of secretory lysosomes seems to be mediated by common molecular machineries regardless of cell type. In the case of genetic immune disorders like Chediak-Higashi syndrome (CHS) and Hermansky-Pudlak syndrome (HPS) type 2, the patients not only have immunodeficiency mainly caused by impaired secretion of LGs by NK cells and CTLs, but also present hypopigmentation (due to impaired melanin secretion) and excessive bleeding (due to absence of dense granules in platelets) (29, 30). In the following sections, we will describe the major components of LGs and their biosynthesis as well as the regulators involved in LG biogenesis.

### 2.1 Major Lytic Granule Components and Their Biosynthesis

#### 2.1.1 Granzymes

Granzymes are a family of serine protease proteins expressed in cytotoxic lymphocytes (4, 31, 32). There are 5 granzyme proteins (A, B, H, K, and M), and each granzyme exhibits unique protease characteristics with different substrate specificities. The wide range of granzyme protease activities induce different apoptosis pathways in caspase-dependent and -independent manners. It is interesting to note that granzyme H and M are predominantly expressed in NK cells (33, 34). However, most of the current understanding of granzymes is based on granzymes A and B. For a detailed description of the characteristics and apoptosis pathway initiated by each granzyme, the reader is referred to these excellent reviews (4, 31, 35).

**Figure 1:**
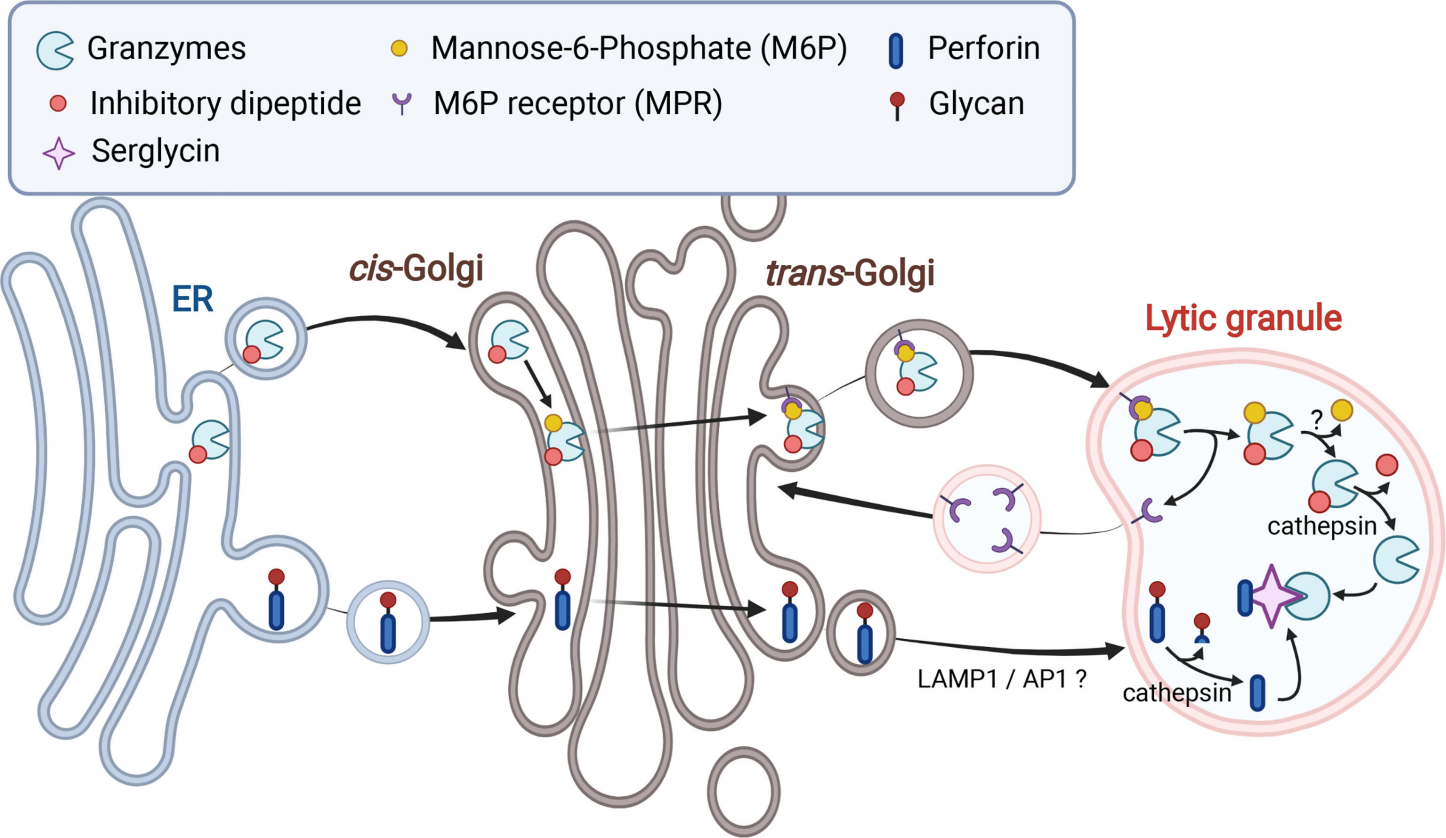
Biosynthesis and trafficking of granzymes and perforin to lytic granules. Both granzymes and perforin are translated into the ER and trafficked to the Golgi. Addition of mannose-6-phosphate (M6P) to granzymes facilitates transport of granzymes to lytic granules (LGs) *via* M6P receptors. Transport of perforin to LGs is mediated by LAMP1 and adaptor protein 1 (AP1) sorting complex *via* an unknown mechanism. Both perforin and granzymes are processed into active forms by cathepsins and other proteases in the LGs but maintained in an inactive state *via* their association with serglycin.

Granzymes are synthesized as pro-enzymes (zymogen), which contain a signal peptide that directs them to the endoplasmic reticulum (ER) and an inhibitory dipeptide that keeps the protein in an inactive form (**Figure 1**) (36). Once the zymogen protein is translated into the ER lumen, it is transferred to the *cis*-Golgi, where it is further modified to have a mannose-6-phosphate (M6P) moiety. The modified zymogen protein is then delivered to the endosome by the M6P receptor (MPR) and finally to the LGs (37). Once in the LGs, granzymes are finally converted to their mature and active form by removal of the inhibitory dipeptide by the cysteine proteases cathepsin C or H (38–40). The importance of granzyme processing is revealed in Papillon–Lefèvre syndrome (PLS), which is caused by autosomal recessive mutation of *CTSC* gene that encodes cathepsin C (**Table 1**) (41, 42). Cathepsin C is a lysosomal cysteine protease that processes granzyme A and B (43). PLS patients are unable to synthesize fully mature and active granzyme due to loss of cathepsin C function and this results in impaired NK cytotoxicity and increased susceptibility to viral infections (38).

**Table 1:** Human Primary Immunodeficiency Syndromes Associated with Impaired Lytic Granule (LG) Degranulation by NK Cells.

NK Cytotoxicity Process	Primary Immunodeficiency	Gene Mutated	Protein Affected	NK Cell Defects in Cytotoxicity
**Lytic Granule Biogenesis**	Papillon–Lefèvre syndrome (PLS)	*CTSC*	Cathepsin C	Impaired maturation of granzymes leading to impaired cytotoxicity
	Familial hemophagocytic lymphohistiocytosis type 2 (FHL2)	*PFR1*	Perforin	Normal LG degranulation but impaired cytotoxicity due to absence of the pore-forming molecule
	Hermansky-Pudlak syndrome type 2 (HPS2)	*AP3B1*	β3A-subunit of adaptor protein 3	Impaired cytotoxicity with enlarged LGs
	Chediak-Higashi syndrome (CHS)	*CHS1/LYST*	CHS1/LYST	Enlarged LGs and impaired cytotoxicity due to defective degranulation (enlarged LGs failed to pass through actin mesh at the CS? Impaired LG polarization?)
**Cytoskeletal regulation**	Wiskott-Aldrich Syndrome (WAS)	*WASP*	WASP	Impaired adhesion, reorganization of F-Actin, and LG polarization
	WASP-interacting protein (WIP) deficiency	*WIPF1*	WIP	No detectable WASP with reduced expression of NK cell activating receptors
	Dedicator of cytokinesis 8 (DOCK8) deficiency	*DOCK8*	DOCK8	Impaired adhesion, reorganization of F-Actin, and LG polarization
	Dedicator of cytokinesis 2 (DOCK2) deficiency	*DOCK2*	DOCK2	Defective RAC1 activation, CS formation, F-Actin reorganization, and impaired degranulation
	Coronin 1A deficiency	*CORO1A*	CORONIN 1A	Impaired reorganization of F-Actin at CS impairing degranulation
**Lytic Granule Traficking**	MYH9-related disease (MYH9-RD)	*MYH9*	Myosin9 Myosin IIa heavy chain	Normal conjugate formation, LG convergence, and MTOC polarization but impaired cytotoxicity due to defective lytic granule movement along F-actin at CS
**Lytic Granule Fusion with the Membrane**	Griscelli Syndrome type 2	*Rab27a*	Rab27a	Impaired cytotoxicity and degranulation due to defective lytic granule docking at the membrane
	Familial hemophagocytic lymphohistiocytosis type 3 (FHL3)	*UNC13D*	Munc13-4	Impaired degranulation of docked lytic granules due to impaired LG tethering to membrane
	Familial hemophagocytic lymphohistiocytosis type 4 (FHL4)	*STX11*	Syntaxin 11	Impaired degranulation due to defective LG priming and SNARE complex assembly
	Familial hemophagocytic lymphohistiocytosis type 5 (FHL5)	*STXBP2*	Syntaxin binding protein 2	Impaired degranulation due to defective LG priming and SNARE complex assembly

#### 2.1.2 Perforin

Perforin is a pore-forming protein that enables delivery of apoptosis-inducing serine proteases like granzymes into target cells (4, 44). The perforin-mediated pores also impose osmotic stress on the target cells inducing apoptosis. This pore-forming activity of perforin is calcium- and pH-dependent; perforin is inactive in an acidic environment (44, 45). Perforin binds to the target cell membrane in a calcium-dependent manner (mediated by a calcium-binding C2 domain), oligomerizes into a pore complex, and creates a pore mediated by the membrane attack complex-perforin (MACPF) domain (46). The indispensable role of perforin activity in NK cells and CTLs is exemplified in type 2 familial hemophagocytic lymphohistiocytosis (FHL2) (**Table 1**) (47, 48). FHL2 is an autosomal-recessive disorder caused by mutation in *PFR1* gene, which encodes perforin. Various mutations affecting the maturation, folding, membrane binding, and oligomerization of perforin have been identified, which causes a highly variable perforin protein expression in patients. Although FHL2 patients presented with normal ranges of other components of LGs as well as normal degranulation processes, patient NK cells have defective cytotoxicity due to an inability to form pores on the bound target cells.

Like granzymes, perforin is initially synthesized as an inactive precursor in the ER and trafficked to the Golgi and finally to the LGs (**Figure 1**) (44, 45). However, the detailed mechanism by which perforin is sorted from the *trans*-Golgi network into the LGs remains unclear. LAMP1 and adaptor protein 1 (AP1) sorting complex, which are direct interacting partners, seem to mediate perforin trafficking from the *trans*-Golgi to the LGs (49, 50). Both LAMP1 and AP1 complex were shown to be important for NK cell-mediated cytotoxicity. Interestingly, depletion of either LAMP1 or adaptin γ, a subunit of AP1 complex, caused retention of perforin in cation-independent (CI)-MPR-positive *trans*-Golgi-derived transport vesicles (49). During the trafficking process, perforin goes through proteolysis and glycosylation. It was recently shown that N-linked glycosylation at the C-terminal end of perforin prevents perforin oligomerization during its transit to the LGs (51). This glycosylation prevents perforin activity in the ER and the Golgi, where calcium is more sufficient, and the pH is neutral. Upon arrival in the LGs, perforin is processed to become an active form, as the C-terminal end of perforin is cleaved by Cathepsin L and other proteases (51, 52).

#### 2.1.3 Granulysin

Granulysin is a member of the saposin-like protein family expressed in NK cells and the pre-activated CTLs of most mammals excluding rodents (53–55). Granulysin is initially synthesized as a 15-kDa precursor protein, which is further proteolytically cleaved into a 9-kDa active form in the LGs (56). The active form of granulysin exhibits pore-forming activity like other members of the saposin-like protein family and permeabilizes the membranes of tumor cells as well as intracellular microbes including bacteria, fungus, and parasites (53, 57). The disrupted membranes not only induce osmotic lysis of target cells, but also become routes for granzymes to enter target cells and intracellular microbes (58, 59).

#### 2.1.4 FasL and TRAIL

Both FasL and TRAIL are type II transmembrane proteins expressed on the surface of NK cells and CTLs and belong to the TNF superfamily (60–63). As mentioned previously, engagement of each ligand with its cognate receptor (collectively known as death receptors) induces apoptosis of the target cell. Interestingly, although these death receptor ligands induce cytotoxicity in target cells *via* distinct molecular processes from the LG components described above, both proteins were also found to be localized at LGs (64–69). Therefore, expression of these death receptor ligands at the surface of NK cells is achieved by degranulation of LGs (66, 70). In the case of FasL, several studies suggested that FasL is contained within distinct LG vesicles that do not contain cytotoxic proteins such as perforin and granzymes (18, 71–73). In addition, it was also suggested that these LG subsets present different signaling requirements for degranulation and rely on distinct molecular processes for their secretion (18, 74). Future studies are required to better elucidate the identity and molecular regulation of FasL-containing vesicles, and it will be interesting to see whether TRAIL is also stored within the same (or a similar) subset of LG vesicles along with FasL.

Like perforin and granzymes, FasL is initially synthesized in the ER, trafficked to the Golgi, and finally sorted to the LGs. A proline-rich domain at the C-terminal end of FasL was found to be essential in this process by mediating interaction of FasL with various SH3 domain-containing proteins (18, 75). FasL becomes phosphorylated by Src kinases recruited to this proline-rich domain and FasL is also ubiquitinated at lysine residues close to the proline-rich domain (76). Both posttranslational modifications of FasL are necessary for appropriate sorting of FasL to the LGs. The molecular processes mediating TRAIL trafficking to the LGs are currently unknown and await future studies.

### 2.2 Regulators of Lytic Granule Biogenesis

#### 2.2.1 Adaptor Protein 3 Complex

Adaptor protein 3 (AP3) complex is a hetero-tetrameric protein complex, which is involved in the sorting of many lysosomal proteins including LAMP1, LAMP2, and LAMP3 (CD63) from the endosome or *trans*-Golgi network to the lysosome (77, 78). The essential roles of AP3 in LG biogenesis are exemplified by type 2 Hermansky-Pudlak syndrome (HSP2), which is caused by mutations in the *AP3B1* gene (**Table 1**) (79, 80). Mutations in β3A-subunit of AP3 (encoded by *AP3B1*) cause instability of the protein, which leads to the loss of the entire AP3 complex (79, 80). As mentioned previously, HSP2 patients commonly present immunodeficiency, oculocutaneous albinism, and excessive bleeding, implicating impaired functions of cells with secretory lysosomes (81). Because AP3 is ubiquitously expressed, AP3-mediated protein sorting seems to be especially critical in the biogenesis of secretory lysosomes and/or in the sorting of secretory lysosome-specific proteins. Indeed, AP3 was found to mediate the sorting of tyrosinase (the protein required for melanin synthesis) into lysosomes in melanocytes (82). In the case of antigen presenting cells (APCs), AP3 mediates the sorting of CD1b molecules into MIIC compartments (83). AP3 deficiency in HSP2 patients was also found to cause impaired cytotoxicity of both NK cells and CTLs (84–86). It is interesting to note that CTLs from the HSP2 patients contain enlarged LGs (86). However, it remains unclear which specific components are sorted by AP3 into LG and whether AP3 contributes to the biogenesis of the specialized organelle itself.

#### 2.2.2 CHS1/LYST Protein

CHS1/LYST protein is a member of the BEACH (Beige and Chediak) family, which commonly contains a BEACH motif at the C-terminal end (87). Among all BEACH family proteins, which are known to regulate vesicle trafficking, CHS1/LYST protein is specifically involved in the homeostasis of lysosomes in cells with secretory lysosomes (88). This is exemplified in Chediak-Higashi syndrome (CHS), which is caused by mutation of the *CHS1/LYST* gene (**Table 1**). Like HSP2, the patients of CHS present recurrent infections, partial albinism, and prolonged bleeding, suggesting impaired activities of cells with secretory lysosomes (87, 89). As expected, both NK cells and CTLs from CHS patients present impaired cytotoxic activities with failure to secrete LGs. However, degradative functions of lysosomes in cells with secretory lysosomes as well as synthesis, processing, and sorting of perforin and granzymes into LGs in CTLs were found to be normal (87, 90, 91). Interestingly, NK cells, CTLs, and melanocytes from CHS patients contain abnormally enlarged lysosomes (87, 90–93). It was shown that the LGs gradually fuse together to become enlarged lysosomes in CTLs (90, 92). In the case of NK cells, CHS1/LYST-depleted or CHS patient NK cells were recently found to have abnormal endolysosomal compartments (91, 93). These observations suggest that the CHS1/LYST protein might mediate lysosome fusion/fission during the lysosomal maturation process. Regarding cytotoxicity, although one study reported that the smaller size of the cortical actin mesh at the CS relative to the enlarged LGs prevented degranulation in *CHS1/LYST*-deficient NK cells (93), important roles of CHS1/LYST in LG polarization to the CS have also been suggested (91). In addition, Mauve, the *Drosophila* homolog of CHS1/LYST, not only regulates vesicle fusion of yolk granules (the secretory lysosomes of the *Drosophila* embryo) but was also found to regulate microtubule nucleation from the microtubule organizing center (MTOC) (94). Therefore, future studies are required to better elucidate the molecular details by which CHS1/LYST regulates the lysosomal fusion/fission process and the impact on cytotoxic activity.

### 2.3 How do NK Cells Protect Themselves From Activities of Synthesized Lytic Granule Contents?

As we have seen so far, each LG component has its own cytolytic activity. This can potentially cause self-destruction of NK cells during synthesis and maintenance of LGs. NK cells have several protection layers to ensure safe storage and trafficking of cytolytic contents until degranulation. First, the acidic environment inside of LGs prevents the activity of the cytolytic proteins. In this low pH environment, perforin and granzymes also interact with chondroitin sulfate proteoglycan known as serglycin (**Figure 1**). The association with serglycin keep both cytolytic proteins in an inactive state until secretion (95–98). In addition, several perforin-specific protection mechanisms have been identified (99). As previously mentioned, perforin is N-linked glycosylated at the C-terminus in the ER (**Figure 1**). This prevents perforin oligomerization and pore-forming activity during its transit to LGs, regardless of calcium concentration and pH (51). Upon arrival at the LGs, the mature perforin without the inhibitory C-terminus is still kept inactive due to very limited availability of calcium (100). In addition, interaction of calreticulin with perforin in the ER and LGs was also suggested to contribute to the inhibition of perforin activity (101).

### 2.4 Remaining Questions on Lytic Granule Biogenesis

Our current understanding of the biogenesis of the LGs is mainly focused on the biosynthesis and sorting of cytolytic proteins into the LGs but not on the LG organelle itself. Are LGs derived from pre-existing lysosomes or are they generated independently from lysosomes? In addition, components of the lysosomes and LGs are often mediated by the same trafficking and sorting machineries. Therefore, it remains unclear how cells containing LGs distinguish cargoes between the two organelles. In this regard, it is interesting to note that proteins like AP3 and CHS1/LYST involved in LG biogenesis and/or LG protein sorting are ubiquitously expressed. Therefore, it would be interesting to elucidate how the mutations in these proteins only impact cells with secretory lysosomes. It was also recently shown that LG size and contents are associated with the efficiency of NK cell cytotoxicity (102). Future studies aimed at elucidating the mechanisms by which NK cells regulate the amount of cytolytic contents and the size of LGs will also be of interest. Finally, we have very limited understanding of the heterogeneity of the LGs. Thus, it will be interesting to examine potential differences among the LGs inside a single NK cell and define not only how these distinct LGs mature but also the signaling mechanisms regulating their exocytosis.

## 3 NK Cell Activating Signaling Leading to Cytotoxicity

To date, dozens of NK cell receptors have been identified which can be classified as inhibitory or activating depending on the signaling pathways engaged by the cytoplasmic tail of the receptor or receptor-associated transmembrane signaling adaptor molecules such as DAP10, DAP12, CD3ζ and FcεRIγ. Although we will not be exhaustively discussing inhibitory and activating receptor signaling in this review, it is important to point out, at a high level, that NK activating receptors such as NKG2D/DAP10, NKp46/CD3ζ, CD94/NKG2C/DAP12 and FcγRIIIA/FcεRIγ/CD3ζ regulate an overlapping set of signaling pathways that culminate in the cytokine production and cell-mediated killing through the directed secretion of LGs.

**Figure 2:**
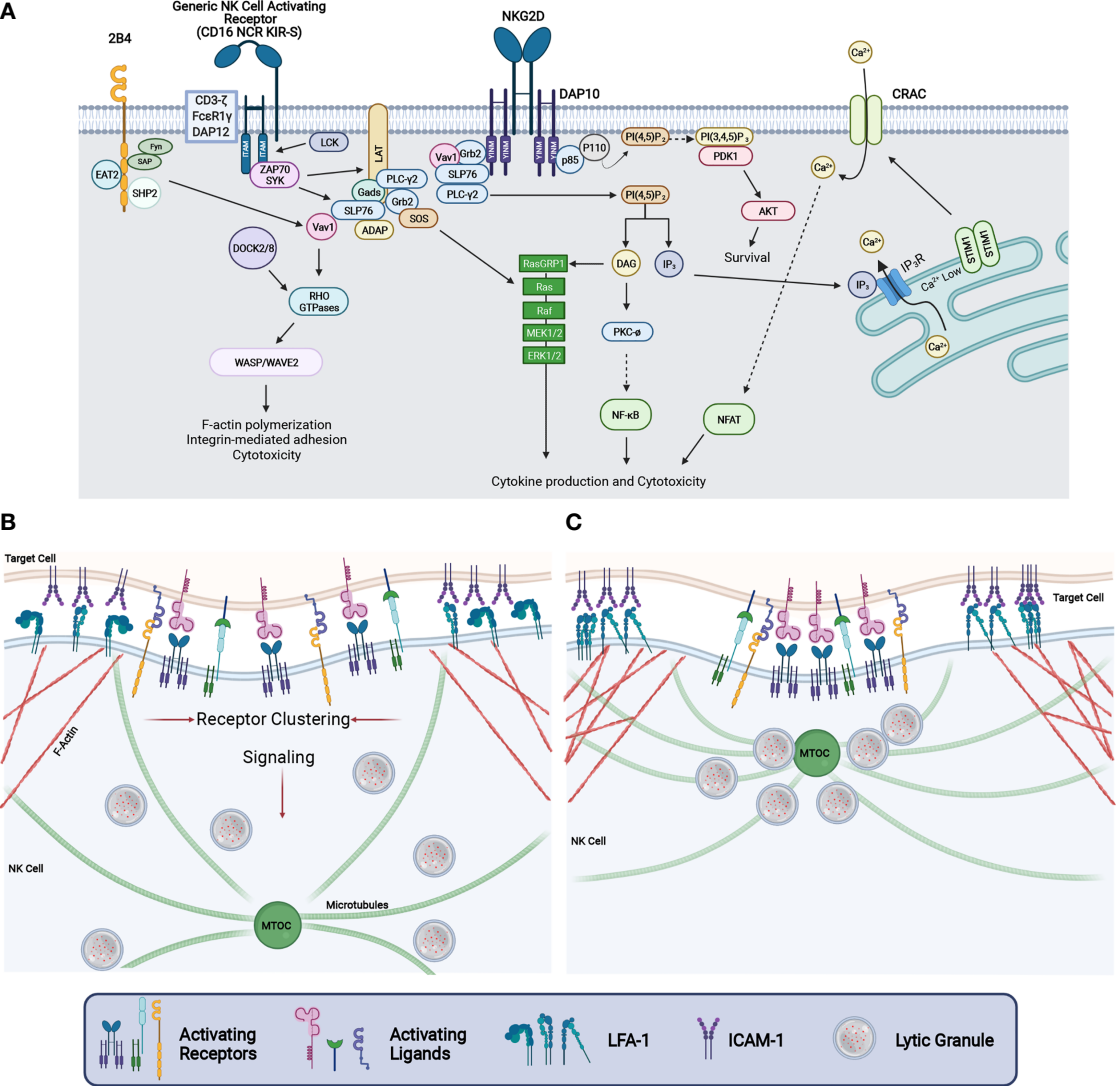
NK cell signaling and cytotoxic synapse maturation. **(A)** Signaling diagram depicting events downstream from human NKG2D-DAP10, 2B4, and NK cell activating receptors coupled to the ITAM containing adaptor proteins CD3-ζ, FCϵR1γ, or DAP12. Ligation of these receptors causes VAV1, SLP76, and PLCy2 phosphorylation which results in the activation of NFAT through calcium release, NFκB activation, and activation of the MAP Kinase cascade. This ultimately leads to increased integrin-mediated adhesion, F-actin reorganization, cytokine production, and cytotoxicity. **(B)** Upon the binding of a target cell, signaling through NK cell activating receptors results in the clustering of receptors while simultaneously enhancing adhesion through integrin affinity maturation and directing LG convergence to the MTOC. **(C)** As the CS matures, activating receptors are clustered at the central region of the CS whereas F-actin and integrins accumulate in the peripheral region of the CS to stabilize adhesion between the NK and target cell. Further signaling from NK activating receptors drive LG convergence and MTOC polarization.

At the pinnacle of signaling from NK activating receptors is the Src family kinase Lck, which directly phosphorylates the YINM motif in DAP10 and the immunoreceptor tyrosine-based activation motifs (ITAMs) within DAP12, CD3ζ and FcεRIγ (**Figure 2A**). In the case of NKG2D/DAP10, phosphorylation of DAP10 by Lck leads to the recruitment of p85/PI3K and Grb2/Vav1 complexes, which then mediate downstream signaling. In contrast, tyrosine phosphorylation of ITAMs by Lck leads to the recruitment of either ZAP70 or SYK tyrosine kinases which subsequently tyrosine phosphorylates other signaling molecules including adaptors and enzymes to promote signaling leading to cytokine production and cytotoxicity (**Figure 2A**). The Src family member Fyn is also involved through its phosphorylation of the immune tyrosine-based switch motif (ITSM) found in the co-stimulatory molecule 2B4. This phosphorylation event further enhances signaling pathways engaged by other activating receptors and includes the phosphorylation of Vav1 and PLCγ2 (**Figure 2A**). PLCγ2 is critically involved in NK cell cytotoxicity and cytokine production as it is the key producer of two second messengers through the cleavage of PI (4, 5)P_2_ located in the inner leaflet of the plasma membrane to diacyl glycerol (DAG) and IP_3_. While DAG participates in the activation of PKC – NFκB and Ras-MAPK pathway activation, IP_3_ stimulates the endoplasmic reticulum to release it luminal store of Ca^2+^ by binding to the ER-localized IP_3_ receptor, which in turn leads to STIM interaction with the calcium release activated calcium (CRAC) channel leading to an influx of extracellular calcium into the cell (**Figure 2A**). This rise in intracellular Ca^2+^ impacts various cellular processes including the activation of various enzymes, proteins involved in F-actin cytoskeletal dynamics and the activation of the transcription factor NFAT which is involved in interferon-γ gene expression (103, 104). For a more detailed description of the NK receptors and signaling pathways regulated, the reader is referred to several excellent reviews on this topic (104–107).

Reorganization of the F-actin cytoskeleton is a critical step in the development of NK cell-mediated killing. The activation of guanine nucleotide exchange factors such as Vav1 and DOCK2 or DOCK8 lead to the activation of Rho family small GTP-binding proteins (Cdc42, Rac1 and RhoA) which regulate F-actin dynamics through the regulation of WASP and WAVE2. F-actin regulation in NK cells is critical to many steps in the development of cell-mediated killing including organization of the CS, activating receptor clustering within the central region of the synapse, integrin-mediated adhesion, lytic granule convergence and the transit of lytic granules to the site of NK – target cell contact to name a few (**Figures 2A–C**) (108–114). Significantly, mutations in genes whose protein products are involved in the regulation of F-actin cytoskeletal dynamics including DOCK2, DOCK8, WASP, WIP and CORONIN-1A are associated with primary human immunodeficiency syndromes resulting from defective F-actin reorganization, cell adhesion and LG release (1, 113) (**Table 1**). Finally, signaling from activating receptors leading to lytic granule convergence and MTOC polarization to the CS are critical to the directed delivery of the lethal LG contents to the contact between the NK cell and its target. In the sections below, we will describe in greater detail the proteins and signaling pathways that regulate lytic granule trafficking and MTOC polarization during NK cell – target cell engagement as well as the final steps involved in LG fusion with the NK cell plasma membrane.

## 4 Lytic Granule Trafficking

### 4.1 Lytic Granule Convergence

**Figure 3:**
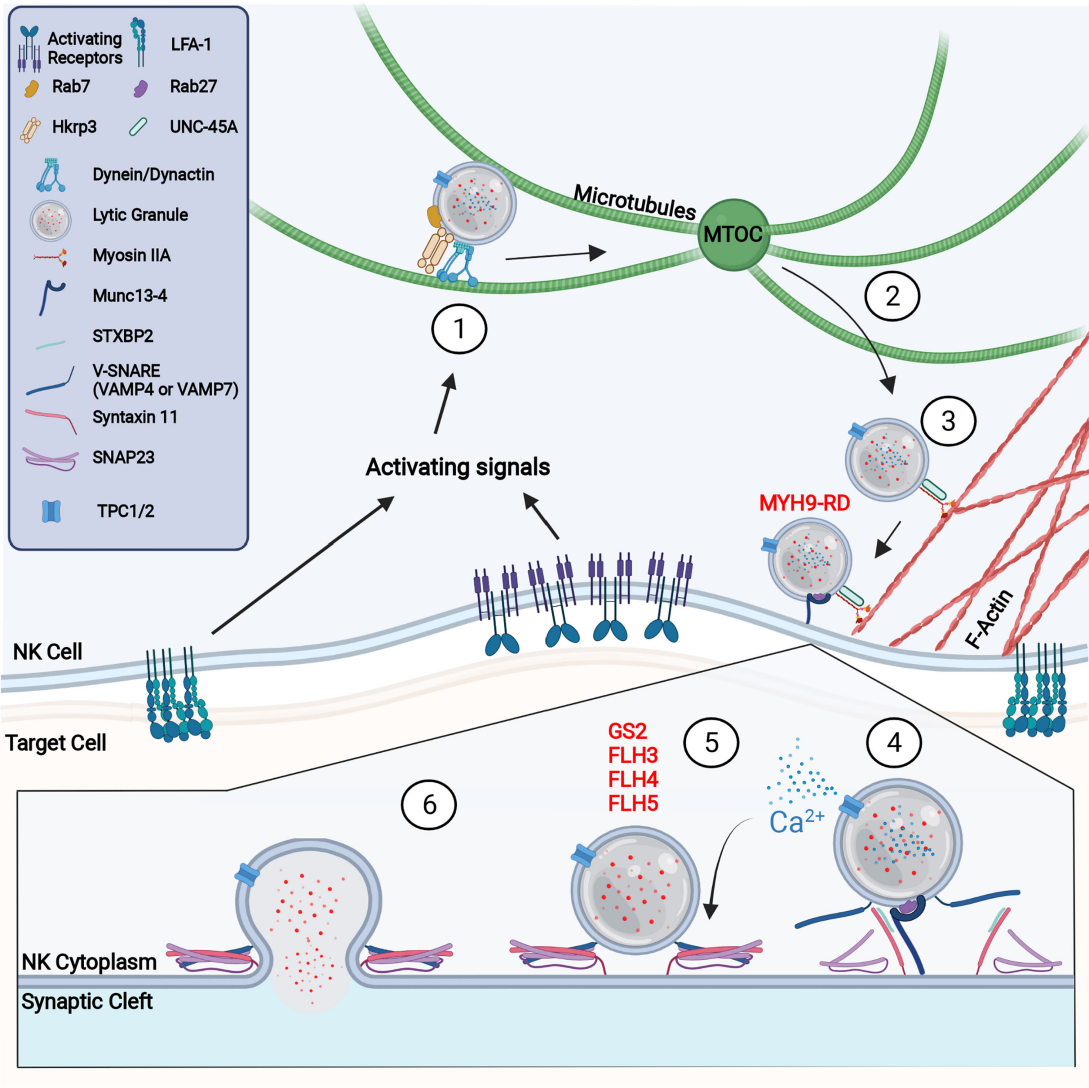
Molecular process of NK cell degranulation (1) NK activating receptor and integrin signaling promotes LG convergence at the MTOC through the activity of the dynein/dynactin complex. (2) Upon further cell stimulation, the MTOC polarizes to the synapse where lytic granules are offloaded onto the F-actin network. (3) Trafficking along F-actin requires the activity of myosin IIA and UNC-45A. Defects in the myosin heavy chain, MYH9, prevents lytic granule penetration of the F-actin network and causes MYH9-related disease (MYH9-RD). (4) Upon reaching the membrane, Rab27a and Munc13-4 dock and tether lytic granules to the CS. Griscelli syndrome type 2 is caused by defects in Rab27a, which results in lytic granules accumulating at the membrane without docking. It is likely that at this step or at prior steps, the NAADP-mediated release of Ca2+ from the LG *via* TPC1 or TPC2 occurs to provide a local accumulation of calcium. Munc13-4 primes lytic granules for release through interaction with Syntaxin 11. (5) STXBP2 mediates formation of the SNARE complex, consisting of Syntaxin 11, SNAP23, and VAMP4 or VAMP7. Defects in Munc13-4, Syntaxin 11, and STXBP2 cause familial hemophagocytic lymphohistiocytosis (FHL) types 3, 4, and 5 respectively. (6) Successful formation of the SNARE complex creates a LG plasma membrane fusion pore through which degranulation occurs.

The release of cytolytic granules is accomplished through a heavily regulated stepwise process beginning with the convergence of cytolytic granules to the MTOC (10). This process occurs rapidly and is initiated through the engagement of adhesion receptors, such as the leukocyte function associated antigen-1 (LFA-1), in combination with other activating NK cell receptors. The function of convergence is both to prepare the LGs for directed secretion to a target cell and to effectively concentrate LGs for enhanced delivery (115, 116). This minimizes off target effects of LG secretion and ensures sufficient delivery of the cytolytic contents. Interestingly, LG convergence occurs in both activating and inhibitory NK CS and is independent of PI3K, MEK, and PLCγ activation, although these signals are required for maturation of the NK CS and degranulation (117). LG convergence also occurs prior to microtubule or F-actin reorganization as Taxol, cytochalasin D, and latrunculin A inhibited MTOC polarization to the synapse but not LG convergence (118, 119). This suggests that LG convergence is an early event downstream of adhesion and prior to large cytoskeletal reorganization events. Interestingly, high dose IL-2 can induce LG convergence independent from adhesion (117). This was found to be dependent on Src kinase activity, which is induced by IL-2 through a non-canonical, JAK3-independent, pathway and is also downstream of LFA-1 activation (**Figure 3**) (117, 120). LG convergence, therefore, rapidly occurs downstream of activation but prior to a commitment to cytotoxicity.

The rapid accumulation of LGs at the MTOC is dependent on dynein/dynactin mediated minus-end-directed movement along the microtubule network (**Figure 3**) (118). Although the dynein/dynactin complex is constitutively localized with LGs in NK cells, dynein-mediated LG movement requires additional adaptor proteins (118, 121). For example, HkRP3, which is localized at LGs and interacts with the dynein/dynactin complex, was found to regulate dynein complex-mediated LG convergence (122). Interestingly, Grb2 interacts with Src and the P150^Glued^ subunit of dynactin which could link Src activation to dynactin signaling (118). This alternative pathway of Src kinase-dependent LG convergence may help explain how high-dose IL-2 can rescue the phenotype of WASP deficiency through the activation of the WASP family member WAVE2 (123–125). Another mechanism that might regulate dynein function is its potential interaction with, and recruitment by, Rab7a and Rab Interacting Lysosomal Protein (RILP) (**Figure 3**) (122, 126). Rab7a was identified in the lysosome fraction of the NK cell line YTS (127) and, with RILP, recruits dynein/dynactin complexes to lysosomes (128, 129). Furthermore, overexpression of RILP in CTLs causes clustering of LGs and prevents plus-end-directed movement, suggesting an important role in LG minus-end trafficking (130). However, the precise mechanisms regulating dynein-directed NK cell movement, and the role of Rab7a, have yet to be fully elucidated.

### 4.2 MTOC Polarization to the NK Cytotoxic Synapse

LG convergence is a prerequisite for the polarization of LGs to the NK CS (131–133). This is accomplished through the polarization of the MTOC and converged LGs to the maturing CS through mechanisms that include F-actin reorganization and continued signaling through clustered receptors (**Figure 3**) (118, 133). Although there are differences in the rate of LG convergence and MTOC polarization between CTLs and NK cells, the mechanisms that control these processes are thought to be similar (134). Indeed, many studies investigating synapse formation and microtubule dynamics performed in the CD4^+^ Jurkat T cell line may be extrapolated, with care, to CD8^+^ T cells and NK cells, despite the lack of cytolytic ability in Jurkat cells. In CTLs, two mechanisms for MTOC polarization have been proposed. The first mechanism is a dynein-dependent cortical sliding mechanism where dynein, anchored to the cell cortex, pulls on microtubules to bring the MTOC toward the synapse (115, 135, 136). This method is supported by the role of adhesion and degranulation promoting adaptor protein (ADAP) in microtubule anchoring and the previous observations of synaptic microtubule anchoring in MTOC movement (135). The second proposed method of MTOC polarization is a capture-shrinkage mechanism where anchored dynein pulls on microtubules which depolymerize, effectively pulling the MTOC to the synapse (135, 137). This mechanism is also plausible as Taxol, which stabilizes microtubules thus preventing depolymerization, and ciliobrevin, which inhibits dynein activity, abrogated MTOC polarization in Jurkat T cells when used together, whereas use of Taxol alone only slowed polarization (135). Additionally, it was recently demonstrated that the kinesin-4 family member KIF21B, regulates microtubule organization and growth by inducing microtubule pausing and depolymerization (138). Knockout of KIF21B in Jurkat T cells resulted in decreased synaptic MTOC polarization attributed to overgrown microtubules at the synapse. MTOC polarization and microtubule organization was rescued by low dose vinblastine, which induces microtubule depolymerization (138). Although, Hooikaas et al. do not believe KIF21B directly participates in dynein-driven capture-shrinkage, they do not exclude the indirect impact excessively elongated microtubules may have on this process. While capture-shrinkage may be the predominant model when the MTOC and CS are diametrically opposed, when modeled with cortical sliding, the two mechanisms appear to work in synergy suggesting that a mechanistic combination may be more appropriate and applicable to a wider variety of interactions (139).

Furthermore, it was observed that MTOC polarization in CTLs appeared to occur through a two-step mechanism where LGs rapidly polarized to the synapse before slowing down to complete their journey (135). This was proposed to occur through the initial localization and function of dynein at the central region of the CS (central SMAC) followed by dynein activity at the pSMAC (140). The specific mechanisms regulating NK cell MTOC polarization remain unclear and warrant further investigation. In CTLs, it has been suggested that the strength of TCR signaling may regulate the specific mechanisms of MTOC polarization (131, 141). How this translates to NK cell signaling is unknown, especially as it relates to strength of signaling emanating from NK activating receptors.

Several cytoskeletal regulatory proteins are known to be critical for NK cell MTOC polarization including the small GTPase CDC42. CDC42 and WASP localize to the MTOC after LG convergence and are required for polarization (10, 142). This is mediated by CDC42 Interacting Protein (CIP4) which couples both the actin and microtubule networks through binding tubulin, CDC42, and WASP. In activated NK cells, CIP4 localizes with the MTOC to the NK CS and could function to anchor the MTOC through WASP or CDC42 activation (142). Furthermore, CIP4 is not required for F-actin accumulation at the synapse suggesting its primary role is on the LGs (142). ADAP, another cytoskeletal regulatory protein could also help apply force to the MTOC, as it is required for insertion of the microtubule plus-end into the ring-like F-actin network at the peripheral region of the CS, known as the peripheral supramolecular activation cluster (pSMAC) (10, 136, 143). Although ADAP is required for CTL degranulation, its role in NK cells is less clear with some reporting that it may be dispensable for NK cell killing (144).

### 4.3 Trafficking at the Cytotoxic Synapse

After MTOC polarization toward the CS, the clustered LGs need to navigate the dense F-actin network at the cell cortex to dock and fuse with the NK cell membrane (145, 146). Although LGs can undergo kinesin-1 plus-ended microtubule movement (147), the proximity of the polarized MTOC and LGs to the synapse is likely sufficient to offload the LGs onto the F-actin network in CTLs (132). Indeed, LG trafficking at the CS has been shown to be independent of plus-ended microtubule movement, as overexpression of RILP kept LGs clustered at the MTOC, therefore preventing plus-ended trafficking, without any impact on lysis (132). However, there are some reports of kinesin-1 regulating plus-ended movement in CTLs (148). Interestingly, it was recently demonstrated that Arl8b regulates MTOC polarization in NK cells through its interaction with the kinesin-1 heavy chain KIF5B and SifA and kinesin-interacting protein (SKIP) (149). Silencing of KIF5B, SKIP or Arl8b led to defective MTOC polarization, suggesting that in NK cells, kinesin could regulate LG trafficking at a much earlier cytolytic stage than in CTLs (149). However, the role of kinesin in NK cell degranulation and the specific mechanisms regulating lytic granule transfer to the F-actin network are still unclear and require further investigation. Lastly, in CTLs it was shown that HDAC6, which deacetylates α-tubulin at Lys40 and interacts with kinesin-1 light chain, is required for proper lytic granule migration to the CS (150). Indeed, although CTLs taken from HDAC6-deficient mice showed a decreased MTOC to target cell distance, lytic granules appeared much more diffuse, suggesting a role for HDAC6 in LG trafficking at the CS (150).

Clearances in the cortical F-actin at the cSMAC have been identified in CTLs (132) and NK cells (151), suggesting a role for an actin motor protein to mediate the final stretch of LG trafficking to the membrane. Indeed, the movement of LGs on F-actin has been shown to be dependent on the non-muscle actin motor myosin IIA (**Figure 3**). Myosin IIA is a hexamer consisting of two heavy chains, two regulatory light chains, and two essential light chains (152, 153) and is constitutively associated with LGs as single molecules rather than a filament (154). This association with LGs could be mediated through direct recognition of phosphatidylserine, binding of Rab27a, or through binding of the WASP/WIP complex (153). Inhibition or depletion of the myosin IIA heavy chain, MYH9, prevents degranulation but does not impair conjugate formation, LG convergence, synaptic actin reorganization, or MTOC polarization (153, 155, 156). The importance of myosin IIA in NK cell function can be fully appreciated in a group of diseases, now referred to MYH9-related disease (MYH9-RD), caused by mutations in the heavy chain MYH9 (**Table 1**) (157, 158). Patients with a truncation in MYH9 had ablated cytotoxicity with intact conjugate formation, MTOC convergence, and MTOC polarization (153). Interestingly, the truncation affected both a region of MYH9 important for cargo binding and removed a constitutively phosphorylated serine (MYH9 S1943) required for MYH9 recruitment to LGs (154). Disruption of this key residue resulted in LGs that were present at the synapse but unable to penetrate the F-actin network to reach the membrane (154).

The interaction of myosin IIA with LGs is also dependent on the chaperone protein UNC-45A. UNC-45A colocalizes with LGs in both resting and activated NK cells and polarizes with the LGs to the NK CS upon target engagement (**Figure 3**) (159). Like MYH9 deficiency, depletion of UNC-45A did not impair conjugate formation, LG convergence, or MTOC polarization but is critical for degranulation (159). Depletion of UNC-45A reduces myosin IIa binding to F-actin without impacting myosin IIA expression or stability (159). In addition to regulating myosin IIA, UNC-45A could have an additional independent role in regulating LG priming, docking and fusion, however, this has not been fully elucidated in NK cells (160).

## 5 Fusion of Lytic Granules With the Membrane and Degranulation

### 5.1 Lytic Granule Docking

After transport to the synapse, cytolytic granules dock at the membrane and are prepared for release. This is mediated through the small GTPase Rab27a which was first identified to play a critical role in degranulation through the study of Griscelli syndrome (GS) patients (**Figure 3**) (161). GS is a rare autosomal recessive disease characterized by partial albinism due to defective melanosome transport. Although originally described as being caused by mutations in myosin Va (GS type 1), it was discovered that mutations in Rab27a (GS type 2) is the predominant disease etiology and is responsible for all GS cases with hemophagocytic lymphohistiocytosis (**Table 1**) (161, 162). Interestingly, loss of Rab27a but not myosin Va resulted in defective CTL and NK cell degranulation and cytotoxicity (161). This degranulation defect was recapitulated in the mouse model of GS type 2, the *ashen* mouse, where it was observed that LG convergence and MTOC polarization was intact (163, 164), however, LG membrane docking was not observed by electron microscopy (165). Additionally, in the absence of stimulation, Rab27a regulates microtubule and actin-dependent LG movement at the cell cortex (166). Rab27a therefore regulates LG movement in unstimulated NK cells and is required for the final stages of NK cell-mediated cytotoxicity. Unsurprisingly, Rab27a is also a key secretory protein required in the degranulation of melanocytes, neutrophils, and pancreatic beta cells, suggesting a similar method of action in the terminal stages of LG export (167–169).

The crucial role of Rab27a in NK cell degranulation is mediated through effector proteins which bind to active GTP-bound Rab27 (170). So far eleven effector proteins have been identified in humans and mice and can be categorized into three distinct groups based on domain composition. The first group is comprised of rabphilin and the synaptotagmin-like proteins (Slp): Slp1, Slp2-a, Slp3-a, Slp4-a, and Slp5. The proteins within this group contain an N-terminal Slp homology domain (SHD), which mediates binding to the switch II region of GTP-Rab27a, and two tandem C-terminal C2 domains (C2A and C2B), which bind phospholipids (170). Slp1 and Slp2-a are expressed in NK cells and CTLs and are thought to mediate LG docking and tethering *via* their C2 domains. However, their role in cytotoxicity is still unclear as CTLs from Slp1- or Slp2-a-deficient mice had intact degranulation (171). Furthermore, expression of the SHD from Slp2-a, which has a dominant negative effect, only resulted in a partial reduction in cytotoxicity suggesting that there might be other important proteins in complex with Rab27a.

The next group of GTP-Rab27a effectors is characterized by the presence of the N-terminal SHD and the distinct absence of C-terminal C2 domains (170). This group is comprised of Noc2 and the Slp homolog lacking C2 domain (Slac2) proteins: Slac2-a, Slac2-b, and Slac2-c. However, no role for Slac2 proteins has been identified in NK cells. The last group contains the protein Munc13-4 which has an N-terminal C2 domain followed by a Rab binding domain (RBD), a MUN domain, and a second C2 domain (170). Interestingly, Munc13-4 lacks a C1 domain, which mediates DAG binding, found in other Munc13 family members and the mechanism of Rab27 binding by the Munc13-4 RBD is uncharacterized despite the importance of Munc13-4 in LG exocytosis. In addition to the Rab27a interactors described above, proteins involved in Rab27 prenylation, as well as several proteins known to bind GDP-bound Rab27 including CORONIN-3, RabGDI, and MAP kinase activating death domain (MADD) have remained largely uncharacterized as it pertains to their roles in NK cell-mediated killing (170, 172).

### 5.2 Lytic Granule Tethering

The tethering of cytolytic granules at the synapse refers to the process by which LGs make initial interaction with the membrane and are prepared for fusion. This is Rab27a-dependent and is thought to be mediated by Slp1, Slp2, and Munc13-4 (**Figure 3**) (165, 171). Munc13-4 mutations were first identified as the cause of familial hemophagocytic lymphohistiocytosis type 3 (FHL3), where it was observed that mutation of Munc13-4 resulted in impaired CTL degranulation with intact conjugate formation and MTOC polarization (**Table 1**) (165). Upon stimulation, Munc13-4 localizes to the CS in CTLs and strongly colocalizes with LGs (162, 165). Interaction with Rab27a is critical to Munc13-4 function as wild type but not RBD-deficient or RBD mutant Munc13-4 was able to restore degranulation in FHL3 CTLs (173). Interestingly, recruitment or retention of Rab27a and Munc13-4 on LGs is co-dependent as Rab27a is not recruited or retained on granules in FHL3 patients and Munc13-4 recruitment or retention on LGs is impaired in GS type 2 patients (162, 173, 174). In contrast to Rab27a deficiency, however, Munc13-4 is not required for LG docking at the membrane, suggesting its main function is through the tethering of LGs (165). Indeed, both the C2A and C2B domains are required for cytotoxicity as they mediate binding to both lipids and soluble N-ethylmaleimide-sensitive factor activating protein receptor (SNAREs) (175). Munc13-4 may have a similar function to synaptotagmin in neuronal degranulation, as C2 domain binding is calcium-dependent, and thus Munc13-4 might be the calcium sensor that triggers synchronous granule release (176, 177). Interestingly, nicotine acid adenine dinucleotide phosphate (NAADP), a Ca^2+^-mobilizing second messenger is involved in exocytosis through their regulation of the two-pore channel (TPC) 1 and TPC2 which are present on lytic granules (178, 179). It was shown in CTLs that NAADP activates TPCs in a manner that is independent of global Ca^2+^release stimulated by IP_3_ suggesting that a local release of Ca^2+^ facilitates lytic granule vesicle fusion (178) (**Figure 3**). The molecular targets of this local calcium release could be molecules involved in vesicle fusion such as Munc13-4.

Interestingly, low levels of degranulation were observed in GS type 2 deficiency, suggesting Munc13-4 might also partner with other Rab proteins. Indeed Munc13-4 binds to Rab11 and Rab15 regulating recycling endosomes and Weibel-Palade body exocytosis (170, 180, 181). In CTLs, it has been demonstrated that Munc13-4 mediates the fusion of Rab11 positive recycling endosomes with Rab27a positive late endosomes which are then transported to the synapse for release (174, 181). However, this does not seem to occur in NK cells. Despite low levels of perforin in recycling endosomes, Rab11 and other recycling endosome markers are not tightly associated with NK cell synapses. This is further confirmed as Munc13-4 deficiency does not impact release of IFNγ and TNFβ, which is mediated through a Rab11 positive recycling endosome pathway (182), and recycling endosome inactivation does not greatly impact preformed LG release (174).

### 5.3 Lytic Granule Priming

LG priming is mediated by the assembly and recruitment of SNARE complex proteins that facilitate the fusion of the LG with the NK plasma membrane at the CS. This is dependent on Munc13-4, which promotes SNARE complex formation through its interaction with the SNARE protein syntaxin 11 (**Figure 3**) (183, 184). Munc13-4 carries out this function by opening the conformation of syntaxin 11 through the removal of its chaperone protein, syntaxin binding protein 2 (STXBP2), which is required for syntaxin 11 stability and subcellular localization (173, 185, 186). Interestingly, overexpression of syntaxin 11 in NK cells and activated CTLs increased their cytolytic potential (187, 188). Like Munc13-4 mutations, mutations in syntaxin 11 cause FHL4 which presents as a defect in NK cell degranulation with intact synapse formation and LG polarization (**Table 1**; **Figure 3**) (189). This was also recapitulated in the mouse model of FHL4 (184). STXBP2 mutations detrimental to its interaction with syntaxin 11 cause FLH5, which has the same phenotype as FLH4 (**Table 1**) (190, 191). Interestingly defective degranulation in FLH4 and FLH5 can partly be rescued by IL-2 treatment. This is thought to occur through alternate pairing as IL-2 induces expression of syntaxin-3, which replaces syntaxin 11 in FHL4, and STXBP1, which replaces STXBP2 in FLH5 (192). Additionally, syntaxin 7 was shown to be required for CTL degranulation but has not been investigated in NK cells (193). Although the function of alternative pairing was investigated in FLH5 patients, the roles of other syntaxins, such as syntaxin-1 and syntaxin-7, warrant further investigation (194).

Lytic granule priming and SNARE complex assembly was recently shown to be supported by septin filaments (195). Septins are GTP-binding proteins which can be organized into 4 subgroups (represented by septin 1, septin 3, septin 6, and septin 7), and can assemble into hetero-hexamers and hetero-octamers, which can assemble to form complex structures (196). Septin 7 is the only member of its subgroup and is indispensable for septin complex formation. Interestingly, depletion of septin 7 decreases septin 1 and septin 2 expression and impairs NK cell degranulation without impacting conjugate formation or lytic granule accumulation at the CS (195). Despite being concentrated to the cell cortex away from the CS, septin 7 puncta were observed in apposition to lytic granules and were found in the crude lysosomal fraction of NK cells (195). Mass spectrometry analysis of septin 2 crude lysosomal fraction immunoprecipitates revealed associations with lytic granule regulatory proteins including syntaxin 11 and STXBP2, which were confirmed by proximity ligation assay and immunoprecipitation (195). Additionally, depletion of septin 7 or septin stabilization with forchlorfenuron, decreased association of STXBP2 with syntaxin 11. This suggests that septin filaments stabilize SNARE complex assembly and are critical for facilitating syntaxin11 and STXBP2 interaction and ultimately, LG membrane fusion.

### 5.4 Lytic Granule Fusion

The final stage of LG degranulation is the fusion of the LGs with the plasma membrane, which is mediated by formation of trans-SNARE complexes. Due to the number of SNARE protein combinations required to make a complete fusion complex, they have not been fully defined in NK cells (197). In human CTLs, membrane fusion is promoted by STXBP2 which forms a trans-SNARE complex with STX11, SNAP23, and VAMP8 (198). In mice, however, this is mediated by VAMP2 and VAMP8 which colocalize with LGs. Loss of VAMP2 and VAMP8 in mice resulted in impaired degranulation and cytotoxicity (199–201), suggesting other VAMPs cannot compensate for the loss of VAMP2 and VAMP8. Indeed, in NK cells, VAMP4 and VAMP7 are required for NK cell cytotoxicity, while VAMP1, VAMP3 and VAMP8 have limited colocalization with LGs and are therefore likely dispensable for SNARE complex assembly (197, 202). In addition to STX11, STX6 may also play a role in NK cell SNARE complex formation as they interact with VAMP7 and VAMP4, and other syntaxins, like STX4, have been shown to regulate mast cell degranulation (**Figure 3**) (203–206). After SNARE complex formation, a fusion pore between the plasma membrane and NK cells are formed through which degranulation occurs. Interestingly, the size and fusion status of the pore determines the amount of granule content release, which in turn may regulate LG membrane recycling (197). In neuronal cells this has been called the “kiss and run” pathway, although the mechanisms regulating this process in NK cells are unclear, especially in the context of cell-to-cell interactions and warrant further investigation.

## 6 Self-Protection of NK Cells Upon Degranulation

We have seen that the acidic environment and low calcium concentration within the LGs provide protection of NK cells from the activities of cytolytic proteins. However, upon degranulation of LGs into the extracellular environment (where calcium is rich and neutral), the cytolytic contents become fully functional. NK cells achieve selective and efficient degranulation by forming a specialized interface with the conjugated target cells upon formation of the CS. This confined space between NK cells and the target cells, also known as a synaptic cleft, enables NK cells to avoid killing unwanted bystander cells and thus, preventing collateral damage. However, the synaptic cleft exposes the NK cell itself to risk from its own degranulated cytolytic molecules. Secreted perforin can bind the plasma membrane (PM) of both NK and bound target cells *via* hydrophobic interactions and create pores on both cell types. However, autolysis of NK cells or CTLs was found to occur in less than 5% of NK cells during the direct cytotoxicity process (207–209). This suggests that NK cells have protective mechanism(s) that prevent the activities of cytolytic molecules on the source NK cells. One study suggested that cathepsin B exposed to the PM upon cytolytic granule secretion provides protection of NK cells from perforin activity (210). In another study, surface exposed LAMP1 upon degranulation was found to prevent perforin binding to the PM of NK cells (211). However, both protection mechanisms by specific surface membrane proteins do not seem to be exclusive and provide complete protection, since both mechanisms were also found to be dispensable for self-protection under certain circumstances (212, 213). Recently, it was shown that the PM of NK cells and CTLs is composed of high order and densely packed lipids, which prevents perforin binding (209, 213). Furthermore, upon degranulation, the fusion of the LG membrane (which has even higher lipid orders than the PM) to the PM at the CS provides additional protection by acting as a perforin-resistant lipid shield (209). This enables unidirectional attack of perforin specific to the target cell membrane (which generally contains lipid with lower density than NK cells), protecting NK cells from unwanted autolysis.

## 7 Serial Killing of NK Cells

For effective immune surveillance, NK cells need to keep surveying potential target cells and kill as many target cells as needed. In physiological situations like acute viral infections or solid tumor environment where viral loads or tumor cells outnumber NK cells, NK cells need to perform multiple rounds of killing to eradicate surrounding target cells. Indeed, NK cells have been shown to kill multiple target cells in a sequential manner (4, 214–216). For NK cells to achieve serial killing, NK cells need to meet the following requirements: 1) NK cells need to contain sufficient LG contents, and 2) NK cells need to have a mechanism(s) to continuously synthesize and/or refill cytolytic contents. It was recently reported that NK cells degranulate approximately 10-20 LGs (which are about 5-10% of total LGs) to mediate cytotoxicity and minimal release of only 2-4 LGs were sufficient to induce target cell death (102). These observations suggest that a single LG is very effective in inducing cell death and a single NK cell has a capacity to perform multiple rounds of killing. Upon repeated CD16 activation, NK cells presented a gradual decrease in both the intracellular perforin levels and the amount of secreted perforin (217). Interestingly, this reduction in perforin secretion could be restored to its initial level when the NK cells were activated by different activating receptors like NKG2D or NKp30. However, similar restoration was not made when NK cells were stimulated *via* CD16 followed by repeated stimulations *via* NKG2D. These results suggest that the order of NK receptors engaged by specific ligands on the target cells plays an important role in the serial killing activity of NK cells. Upon activation of NK cells by target cells, it was shown that NK cells induce rapid *de novo* synthesis of LGs (218). Interestingly, this rapid biogenesis of LGs was found to originate from endosomal routes instead of budding and maturing from the *trans*-Golgi network. In this regard, NK cells activated by the target cells were found to go through active endocytosis internalizing cytolytic granule components including LAMP1, granzyme B, and MUNC13-4 (219–221). It is important to note that inhibition of the endocytic process of NK cells resulted in a reduction of cytotoxicity. This suggests that endocytosis of cytolytic contents upon degranulation might also be an important process that enables NK cells to perform serial target killing.

Upon delivery of the LGs to the target cell, NK cells need to disassemble the established CS and detach from the target cell. However, compared to the well-established understanding of the initial target recognition and cytotoxicity process, how NK cells determine the termination of the killing and mechanisms behind this detachment process remain elusive. Recently, it was shown that successful cytotoxicity that leads to the death of the target cells is a determinant factor for NK cell detachment (207, 222). Target cells going through apoptosis were found to downregulate NK cell-activating ligands such as MICA, MICB, and B7-H6 as well as adhesion molecules including CD54 and CD102 (222). Along with these events, NK cells also reduce expression of activating receptors upon activation, which might decrease further activation required for cytotoxicity as well as signals necessary for sustained integrin-mediated adhesion (217, 222–224).

Despite the above interesting observations, many important questions on serial killing of NK cells remain. First, a more detailed understanding the of the mechanisms contributing to LG re-generation after each cytotoxic event is needed. Treatment of NK cells with IL-2 or IL-15 was reported to restore perforin and granzyme B levels during serial killing (214). Elucidating the molecular pathways behind the restoration processes will be important. Additionally, defining the replenishment process, which merges both recycled and newly synthesized cytolytic contents to form a complete LG will be an interesting topic. In the detachment process of NK cells, it remains unclear how fast the downregulated NK activating receptors become re-expressed at the normal level. For example, the proteolytic cleavage of CD16 upon activation can be a critical problem in antibody-based anti-cancer therapy, since CD16 expression is required for serial ADCC against tumor cells (217). In this case, it will be important to elucidate the signaling pathway inducing CD16 re-expression and to explore the therapeutic options of using NK cells expressing non-cleavable CD16. Interestingly, it was recently shown that NK cells utilize LG-mediated cytotoxicity for their initial killing events and then switch to death receptor-mediated cytotoxicity (4). The physiological purpose of this phenomenon remains unclear. It is also possible that death receptor-mediated killing (which is slower than cell-mediated cytotoxicity) is revealed in the end as LG is exhausted in NK cells. Regardless, it will be interesting to better define the crosstalk between these cytotoxicity pathways during serial killing. In addition, persistent activation of NK cells can also promote NK cell exhaustion (225–227). Therefore, NK exhaustion during serial killing is also a very important topic in NK cell-mediated therapy.

## 8 Concluding Remarks

The spontaneous cytotoxic activity of NK cells is not only the first line of defense against microbial infections or tumors but is also an ultimate requirement for clearance of these diseases. NK cells eliminate unhealthy/stressed cells by directly secreting apoptosis-inducing molecules toward the target cells. To lyse target cells without any damage on NK cells themselves or healthy bystander cells, NK cell-mediated cytotoxicity is achieved *via* a series of tightly regulated molecular processes. Advances in human genetic research, genome editing, and microscopic technologies combined with diverse fluorescent sensors have enabled us to better elucidate this molecular regulation with improved temporal and spatial resolution. Future advances in uncovering the mechanistic insights of NK cell cytotoxicity will be invaluable to reveal novel therapeutic opportunities to treat primary immunodeficiency syndrome patients with impaired NK cell functions and to improve the efficacy of current approaches in NK cell-based anti-cancer therapy.

## Author Contributions

HH, MM, and DDB wrote and edited the manuscript and generated the figures. All authors contributed to the article and approved the submitted version.

## Funding

This work was supported in part of by NIH grant AI120949 to DDB.

## Conflict of Interest

The authors declare that the research was conducted in the absence of any commercial or financial relationships that could be construed as a potential conflict of interest.

The handling editor declared a past co-authorship with the author DDB.

## Publisher’s Note

All claims expressed in this article are solely those of the authors and do not necessarily represent those of their affiliated organizations, or those of the publisher, the editors and the reviewers. Any product that may be evaluated in this article, or claim that may be made by its manufacturer, is not guaranteed or endorsed by the publisher.
